# Clinical and Biological Significances of a Ferroptosis-Related Gene Signature in Glioma

**DOI:** 10.3389/fonc.2020.590861

**Published:** 2020-11-20

**Authors:** Shenghua Zhuo, Zhimin Chen, Yibei Yang, Jinben Zhang, Jianming Tang, Kun Yang

**Affiliations:** ^1^ Department of Neurosurgery, First Affiliated Hospital of Hainan Medical College, Haikou, China; ^2^ State Key Laboratory of Oncogenes and Related Genes, Renji-Med-X Clinical Stem Cell Research Center, Renji Hospital, School of Medicine, Shanghai Jiao Tong University, Shanghai, China; ^3^ Department of Physical Education, Hainan Normal University, Haikou, China; ^4^ Department of Radiation Oncology, Zhejiang Provincial People’s Hospital, People’s Hospital of Hangzhou Medical College, Hangzhou, China

**Keywords:** glioma, ferroptosis, signature, prognosis, risk score

## Abstract

Ferroptosis is a form of cell death characterized by non-apoptosis induced by small molecules in tumors. Studies have demonstrated that ferroptosis regulates the biological behaviors of tumors. Therefore, genes that control ferroptosis can be a promising candidate bioindicator in tumor therapy. Herein, functions of ferroptosis-related genes in glioma were investigated. We systematically assessed the relationship between ferroptosis-related genes expression profiles and prognosis in glioma patients based on The Cancer Genome Atlas (TCGA) and Chinese Glioma Genome Atlas (CGGA) RNA sequencing datasets. Using the non-negative matrix factorization (NMF) clustering method, 84 ferroptosis-related genes in the RNA sequencing data were distinctly classified into two subgroups (named cluster 1 and cluster 2) in glioma. The least absolute shrinkage and selection operator (LASSO) was used to develop a 25 gene risk signature. The relationship between the gene risk signature and clinical features in glioma was characterized. Results show that the gene risk signature associated with clinical features can be as an independent prognostic indicator in glioma patients. Collectively, the ferroptosis-related risk signature presented in this study can potentially predict the outcome of glioma patients.

## Introduction

Globally, central nervous system (CNS) tumors accounted for 1.6% of all new tumor cases and 2.5% of all cancer-related deaths, in 2018 (1). The incidence of gliomas has been increasing annually. Moreover, the prognosis of gliomas, as a common type of primary CNS tumors, has remained poor, especially in glioblastoma (GBM) patients despite the availability of several treatments including surgery, radiotherapy, and chemotherapy. In addition, some low-grade gliomas (LGGs) are not responsive to current treatments. This calls for the development of effective treatments for glioma patients (2).

In recent years, several studies have investigated tumor ferroptosis. Ferroptosis, an iron-dependent pathway of cell death, differs from new forms of programmed cell death such as apoptosis, pyrolysis, and autophagy. In 2012, it was described that this pathway relies on intracellular iron thus differs from apoptosis, necrosis, and autophagy in terms of morphology, biochemistry, and genetics. Ferroptosis is characterized by the rupture and blistering of cell membranes, mitochondrial shrinkage, increased membrane density, decreased or disappearance of mitochondrial ridges, rupture of outer mitochondrial membranes, as well as normal-sized nuclei without condensed chromatin (3). The system X_C_¯ and glutathione peroxidase 4 (GPX4) in the classical glutathione pathway are the two key regulatory points of ferroptosis regulatory mechanisms. The system X_C_¯ simultaneously accepts input of extracellular cystine and output of intracellular glutamate. In this system, intracellular cysteine is reduced to cysteine, which is the rate-limiting precursor during glutathione biosynthesis. Inhibition of the system X_C_¯ weakens the glutathione biosynthesis and disrupts the redox balance in cells leading to ferroptosis. Also, inhibition of the system X_C_¯ on plasma membranes cause the small-molecule erastin to reduce cellular acquisition of cysteine. Consequently, cysteine impedes the biosynthesis of glutathione, which is a substrate of GPX4, thereby induces the accumulation of reactive oxygen species (ROS) and ferroptosis. ROS accumulation requires iron ions. Studies have indicated that the small-molecule RSL3 can cause ferroptosis by inhibiting GPX4 (4). Several pathways such as mevalonate, iron metabolism, lipid metabolism, glucose metabolism, and iron autophagy pathways are involved ferroptosis (5, 6).

Previous studies have demonstrated strong association of ferroptosis with mammalian neurodegenerative diseases (e.g., Alzheimer’s disease, Huntington’s disease, and Parkinson’s disease), cancer, stroke, traumatic brain injury, ischemia-reperfusion injury, and acute renal failure (7, 8). Recent studies have revealed the role of iron metabolism in cancer stem cells (CSC) and suggested that specific targeting of iron metabolism in CSCs may improve the efficacy of cancer treatment (9). Metabolic reprogramming has been linked to the acquired sensitivity to ferroptosis. The development of effective therapies against tumors that are insensitive to current treatments requires in-depth knowledge of processes that regulate tumor sensitivity. Since tumor cells can maintain or acquire sensitivity to ferroptosis while escaping other forms of cell death, there is increased attention to the development of ferroptosis therapies for tumors (10). The nervous system contains many polyunsaturated fatty acids in the human body, which are the major substrates for the production of peroxides. Thus, targeting ferroptosis can be an avenue for treating gliomas. Similarly, inhibition of autophagy increases susceptibility of GBM stem cells to temozolomide (TMZ) by igniting ferroptosis (11). Further in-depth research is required to understand the mechanism of ferroptosis in gliomas to provide ideas for developing novel drugs against gliomas. It is important therefore analyze the expression patterns of ferroptosis-related genes in the glioma patients, as well as their prognostic values.

Herein, we systematically analyzed the characteristics of ferroptosis-related genes in gliomas based on the CGGA and TCGA RNA-seq datasets, as well as the clinical information. Our findings reveal that ferroptosis-related genes can be used to classify patients with gliomas based on clinical and molecular features. Furthermore, we designed a risk signature containing 25 ferroptosis-related genes for predicting the prognosis of glioma patients.

## Materials and Methods

### Data Collection

The CGGA RNA sequencing (RNA-seq) dataset(mRNAseq_693, mRNAseq_325)and corresponding clinical and molecular information, such as gender, age, grade, subtype, IDH status, 1p/19q status, MGMT promoter status, and survival information, were downloaded from CGGA database (http://www.cgga.org.cn/) as training cohort. Similarly, the TCGA RNA-seq database (https://portal.gdc.cancer.gov/) with 698 glioma samples was obtained as a validation cohort. Thereafter, LGG and GBM RNA-seq data were merged separately, and the batch correction was performed *via* the SVA package. Ferroptosis related genes were obtained from THE HUMAN GENE DATABASE (https://www.genecards.org/) by searching the keywords “Ferroptosis” and other related literature (5, 12). Consequently, the 113 ferroptosis related genes were included in the analysis, and are provided in **Supplementary Table 1**.

### Glioma Subclasses Identification

The ferroptosis-Related genes obtained were subsequently used in non-negative matrix factorization (NMF) clustering (13). A filtering procedure was conducted before performing NMF. Candidate genes with low median absolute deviation (MAD) value (MAD ≤ 0.5) across the glioma patients were excluded. MAD is not only used as a measure of statistical deviation, but also a robust statistic, which provides reliably measures variance than the standard deviation, and can better adapt to the outliers in the data set, and a small number of outliers will not affect the final results. Thereafter, the R package “survival” was used for Cox regression analysis to evaluate the association of all candidate genes with overall survival. Also, the 84 genes with high variance (MAD > 0.5) and significant prognostic value (*P* < 0.05) were selected for sample clustering. Consequently, unsupervised NMF clustering methods were performed using the NMF R package on the metadata set, and the best cluster number was chosen as the coexistence correlation coefficient K value 2. Moreover, T-distributed stochastic neighbor embedding (t-SNE)–based approach was adopted to validate the subtype assignments using the mRNA expression data of above ferroptosis genes.

### Gene Signature Identification and Score Construction

The prognostic value of ferroptosis-related genes in the CGGA training cohort was determined by univariate Cox regression analysis where *P* ≤ 0.001 was considered statistically significant. The prognostic risk characteristics were assessed using “glmnet” (14) and “survival” R package based on the least absolute shrinkage and selection operator (LASSO) method. According to the patient’s clinical information and risk score, independent prognostic factors were selected by multivariate Cox regression analysis. Next, a nomogram was constructed using the survival rate and “RMS” R package, and a correction curve was drawn to evaluate the consistency between the actual and predicted survival rates. Moreover, the concordance index (C index) was calculated, and the value range was 0.5–1.0. Values 0.5 and 1.0 represent random opportunities and excellent ability to predict survival using this model, respectively.

### Statistical Analysis

Patients in CGGA training and TCGA validation cohorts were divided into high-risk and low-risk groups based on the median risk score. Kaplan-Meier survival analysis and 2-sided log-rank test were used to difference in the overall survival between the stratified groups. Univariate and multivariate Cox regression analyses were used to determine independent prognostic factors. ROC curve analysis was used to predict overall survival with R package “pROC”. Student’s *t*-test and chi-square test were adopted to compare differences in pathology and molecular characteristics between different patient groups. All statistical analyses were carried out with the R software, and *P* ≤ 0.05 was considered statistically significant.

### Gene Ontology and Kyoto Encyclopedia of Genes and Genomes

To explore the functional annotation of 25 genes used to establish risk models, the “clusterProfiler” R software package was used to visualize gene ontology (GO) and Kyoto Encyclopedia of Genes and Genomes (KEGG) results (15).

## Results

### Classification of Gliomas Based on Ferroptosis-Related Genes

**Figure 1:**
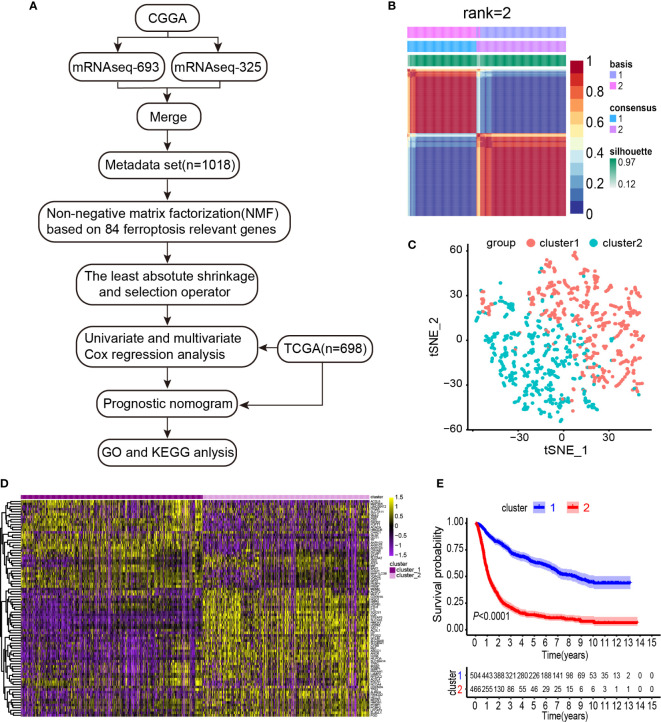
Identification of gliomas subclasses using NMF consensus clustering in the ferroptosis set. **(A)** Flow chart of the study. **(B)** NMF clustering using 84 ferroptosis-related genes. Patients were divided into cluster 1 and cluster 2. **(C)** t-SNE analysis supported the stratification into two gliomas subclasses. **(D)** Heat map of two clusters defined by the ferroptosis-related genes expression. **(E)** Survival analysis of patients in Clusters 1 and 2 in CGGA cohort.

To systematically describe our study, a flow chart was developed (**Figure 1A**). From the CGGA RNA-seq dataset, we obtained 1,018 gene expression profiles of samples and 113 ferroptosis-related genes. A total of 84 ferroptosis-related genes selected based on the MAD value > 0.5; significant prognostic value, *P* < 0.05 were subjected to NMF analysis. The NMF was used to divide glioma samples into 2 different clusters (cluster 1 and cluster 2). The purpose of NMF is to identify potential features in gene expression profiles by decomposing the original matrix into two non-negative matrices (16). A comprehensive correlation coefficient was used to determine the optimal k value. Thereafter, the optimal total cluster number was set to k = 2 (the two subclasses were designated as cluster 1 and cluster 2). For instance, when k = 2, the consensus matrix heat map maintained a clear and sharp boundary, indicating that the samples had stable and robust clusters (**Figure 1B** and **Supplementary Figure 1**). The consensus matrix heat maps are displayed at K values of 3, 4, 5, and 6 (**Supplementary Figure 2**). To verify the subclass distribution, t-SNE was performed to reduce features dimensionality, and it was evident that the subclass names were largely consistent with the two-dimensional pattern of t-SNE distribution (**Figure 1C**). Gene expression heat maps of the two clusters are illustrated in **Figure 1D**. The consensus clustering revealed significant differences in the clinical and molecular features between the two glioma clusters (**Table 1**). However, Chi-square test revealed that cluster 2 patients were significantly associated with primary tumors (58%, *P* < 0.001), GBM histology (64%, *P* < 0.001), high-grade (64%, *P* < 0.001), elderly at diagnosis (62%, *P* < 0.001), IDH wild type (73%, *P* < 0.001), 1p/19q non-codeletion (97%, *P* < 0.001), and MGMT promoter un-methylation (49%, *P* = 0.009). Thus, cluster 2 glioma patients exhibited shorter survival time (log-rank, *P* < 0.0001) as compared with those in cluster 1 (**Figure 1E**). Consistent with this finding, we also observed that cluster 2 has a poorer prognosis in Oligodendroglioma with IDH-mutant and 1p/19q co-deletion, Astrocytoma with IDH-mutant, Astrocytoma with IDH wild type, GBM with IDH-mutant, and GBM with IDH wild type based on the integrated diagnostic guideline of WHO grade 2016 (**Supplementary Figure 3**).

**Table 1:** Characteristics of patients in cluster 1 and 2 in CGGA cohort.

Characteristic	N	cluster 1, N = 532 ^1^	cluster 2, N = 486 ^1^	*p*-value ^2^
**PRS-type**	1,013			<0.001
Primary		372	279	
Recurrent		150	182	
Secondary		9	21	
**Histology**	1,013			<0.001
GBM		78	310	
LGG		453	172	
**Grade**	1,013			<0.001
WHO II		239	52	
WHO III		214	120	
WHO IV		78	310	
**Gender**	1,013			0.3
Female		227	189	
Male		304	293	
**Age**	1,012			<0.001
≤ 41		287	183	
> 41		244	298	
**IDH mutation status**	961			<0.001
Mutant		402	127	
Wild type		83	349	
**1p/19q codeletion status**	938			<0.001
Codeletion		200	11	
Non-codeletion		299	428	
**MGMTp methylation status**	844			0.009
methylated		261	209	
un-methylated		173	201	

^1^Statistics presented: n; median (IQR).

^2^Statistical tests performed: chi-square test of independence; Wilcoxon rank-sum test.

### Construction of Prognostic Gene Signatures Related to Gliomas and Ferroptosis

**Figure 2:**
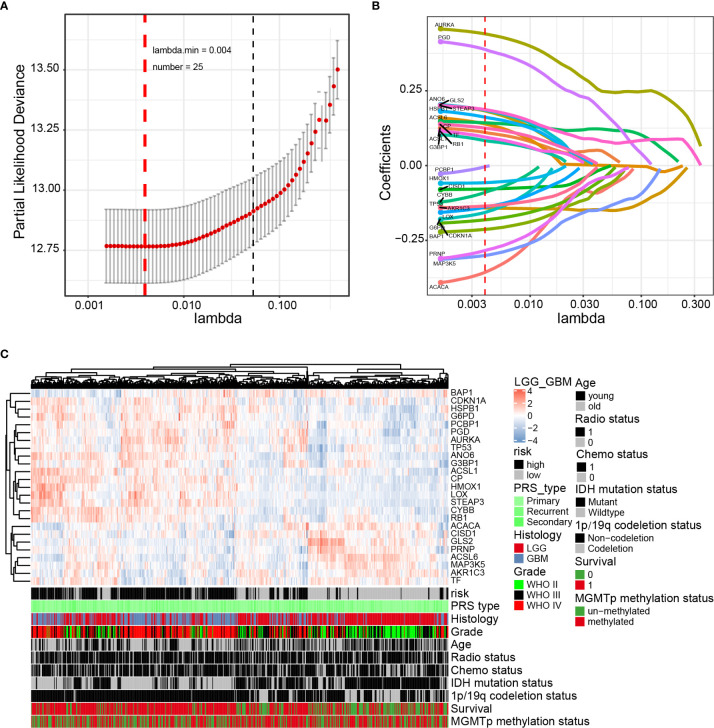
Identification of a 25-gene risk signature for overall survival by LASSO regression analysis in CGGA cohort. **(A)** Cross-validation for tuning parameter selection in the proportional hazards model. **(B)** LASSO coefficient spectrum of 25 genes in gliomas. **(C)** Heatmap shows the association of risk and clinic pathological features based on the 25-gene risk signature. LASSO, least absolute shrinkage and selection operator.

Next, the prognostic role of ferroptosis-related genes in glioma was examined. Among the glioma patients in the CGGA training cohort, 70 overall survival associated genes were identified through the univariate Cox regression analysis (*P* < 0.001) (**Supplementary Figure 4A**). In addition, the multivariate Cox regression analysis identified 25 overall survival associated genes in glioma patients (*P* < 0.05) (**Supplementary Figure 4B**). Biomarkers of the 25 genes were screened using the LASSO regression algorithm (**Figures 2A, B**), which minimizes the risk of overfitting. The patients’ risk scores were calculated from the expression levels and regression coefficients. The results obtained were used to classify patients into low-risk and high-risk groups based on the median risk score. In our study, patients in the high-risk group had primary gliomas, exhibited GBM histology, high-grade, advanced age, IDH wild type, 1p/19q non-codeletion, and MGMT promoter un-methylation (**Figure 2C** and **Table 2**). On the contrary, patients in the low-risk group had primary gliomas, LGG histology, low-grade, young group, IDH mutant type, 1p/19q codeletion (*P* < 0.001), and MGMT promoter methylation (*P* = 0.004).

**Table 2:** Characteristics of patients in low and high-risk scores in CGGA cohort.

Characteristic	N	High-risk, N = 485^1^	Low-risk, N = 485^1^	*p*-value^2^
**PRS-type**	966			<0.001
Primary		274	352	
Recurrent		185	126	
Secondary		23	6	
**Histology**	966			<0.001
GBM		312	62	
LGG		170	422	
**Grade**	966			<0.001
WHO II		42	228	
WHO III		128	194	
WHO IV		312	62	
**Gender**	970			0.3
Female		191	208	
Male		294	277	
**Age**	969			<0.001
≤41		180	260	
>41		304	225	
**IDH mutation status**	921			<0.001
Mutant		132	368	
Wild type		340	81	
**1p/19q codeletion status**	896			<0.001
Codeletion		9	190	
Non-codeletion		433	264	
**MGMTp methylation status**	817			0.002
methylated		210	246	
un-methylated		206	155	

^1^Statistics presented: n.

^2^Statistical tests performed: chi-square test of independence.

### Correlation of Prognostic Risk Scores for 25 Glioma Gene Signatures With Pathological Features

**Figure 3:**
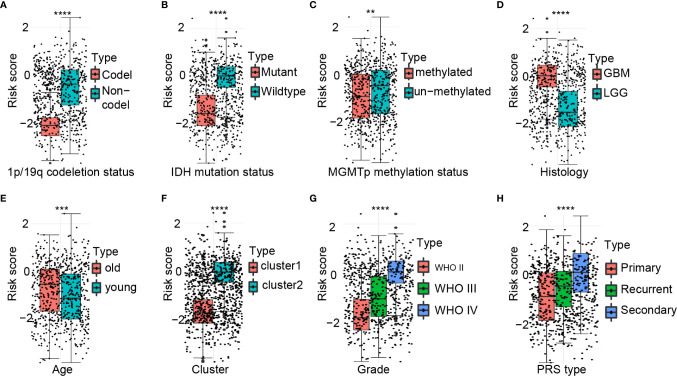
Association between the ferroptosis-related signature and other pathological features in CGGA cohort. **(A**–**H)** Distribution of the risk score in patients stratified by 1p/19q status **(A)**, IDH status **(B)**, MGMT promoter methylation **(C)**, Histology **(D)**, Age **(E)**, Cluster **(F)**, WHO grade **(G)**, and PRS type **(H)**. ****P < 0.0001; ***P < 0.001; **P < 0.01.

There were significant differences in the risk scores between patients with different 1p/19q (*P* < 0.0001), IDH (*P* < 0.0001), MGMT promoter (*P* < 0.01), histological type (*P* < 0.0001), age at diagnosis (*P* < 0.001), cluster group (*P* < 0.0001), WHO grading (*P* < 0.0001), and PRS type (*P* < 0.0001) status (**Figures 3A–H**).

### Survival Analysis of Glioma Prognostic Risk Scores and Correlations With Pathological Features

**Figure 4:**
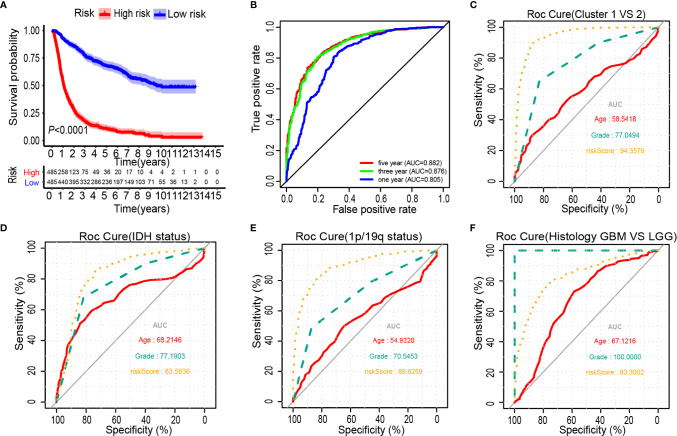
Prognostic significance of the 25-gene signature derived risk scores in CGGA cohort. **(A)** Kaplan-Meier analysis of CGGA gliomas patients was stratified by median risk. **(B**–**F)** High-risk scores are associated with general poor survival of gliomas. ROC curves showed the predictive efficiency of the risk signature, overall survival **(B)**, Cluster 1/2 subgroups **(C)**, IDH status **(D)**, 1p/19q status **(E)**, and Histology **(F)**.

The Kaplan-Meier analysis showed that the overall survival outcome of the high-risk group was worse than that of the low-risk group (**Figure 4A**). The specificity and sensitivity of the risk scores to predict pathological features were determined from the ROC curves by calculating the areas under the curve (AUCs) of the age and grade. Notably, the risk scores showed the potential to predict the survival status (5-year AUC = 0.882), cluster 1/2 (AUC = 0.944), IDH mutation (AUC = 0.836) and 1p/19q status (AUC = 0.896) in the CGGA dataset (**Figures 4B–E**). The risk score of the tumour histological type was higher than that of age (AUC = 0.833) (**Figure 4F**). We also observed that the risk score had prognostic value in gliomas stratified by the integrated diagnosis of WHO grade 2016 (**Supplementary Figure 5**). Similar to the 25-gene signature, TP53 mutation status was also significantly correlated with the prognosis of patients with gliomas (**Supplementary Figure 6A**). To investigate whether the 25-gene signature was independent of TP53 mutation status, patients with gliomas were divided into high- and low-risk groups based on TP53 mutation status. Kaplan-Meier overall survival curves of the two groups based on the 25-gene signature were significantly different in the TP53 wild type and TP53 mutation gliomas cohorts (**Supplementary Figures 6B, C**). To explore whether the 25-gene signature was independent of TP53 mutation type, we performed prognostic analysis of the largest subgroup, which contained TP53 missense mutations. Interestingly, the high-risk group exhibited a shorter overall survival time of glioma patients with TP53 missense mutation (**Supplementary Figure 6D**). Moreover, changes in genetic frequencies (mutation or copy number change) of the 25 genes were very low in gliomas, the genetic variation of TP53 was 39%, that of RB1 was 5%, and that of ACACA was 2%, and the rest were less than 1% (**Supplementary Figure 7**). It shows that the status of these genes is not sufficient to distort the results of risk scores as a prognostic factor. Considering the important biological function of each ferroptosis related genes in the occurrence and development of glioma, the expression of the 25 genes in gliomas with different grades and IDH groups in the CGGA dataset was investigated (**Supplementary Figure 8**). Results showed that only the expression of BAP1 was similar among different grades of gliomas. Except for BAP1 and TP53, there were differences in the expression of 23 genes between IDH wild type and mutant.

### Univariate and Multivariate Cox Analyses of Glioma Prognostic Risk Scores, Survival Distributions, and Heatmaps

**Figure 5:**
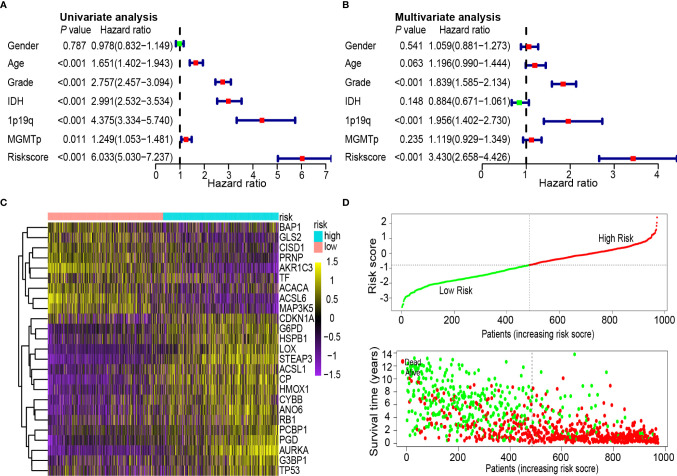
Univariate and multivariate Cox analysis of prognostic risk scores for gliomas, distribution of survival status and heat map. **(A)** Univariate Cox regression analysis. Forest plot of associations between risk factors and the survival of gliomas. **(B)** Multiple Cox regression analysis. The ferroptosis-related gene signature is an independent predictor of gliomas. **(C)** Heat map of ferroptosis-related gene expression profiles in the prognostic signature of gliomas. **(D)** Distribution of risk score and patient survival time, and status of gliomas. The black dotted line is the optimal cut-off value for dividing patients into low-risk and high-risk groups.

To determine whether the 25-gene signature was an independent prognostic indicator, univariate and multivariate Cox regression analyses were performed on CGGA data sets. Univariate Cox regression analysis demonstrated that the risk scores were associated with the overall survival rate of glioma patients (*P* < 0.001). Notably, high-risk scores correlated with poor survival (**Figure 5A**). Multivariate Cox regression analysis revealed that the risk scores were independent risk factors for predicting the overall survival rate of glioma patients (*P* < 0.001) (**Figure 5B**). Differentially expressed ferroptosis-related genes between the high- and low-risk groups in the CGGA database are presented in a heat map (**Figure 5C**). The patients were ranked from left to right according to the risk scores shown at the top of **Figure 5D**. The risk scores increased from left to right. Moreover, at the bottom of **Figure 5D**, patients were ranked from left to right according to risk scores, which showed the survival distribution of each patient. Consecutively, the distribution of survival status and risk scores showed that compared with another group, patients with a risk score of −0.789 or higher generally had poorer survival.

### Validation of CGGA Database Risk Scores by the TCGA data

**Figure 6:**
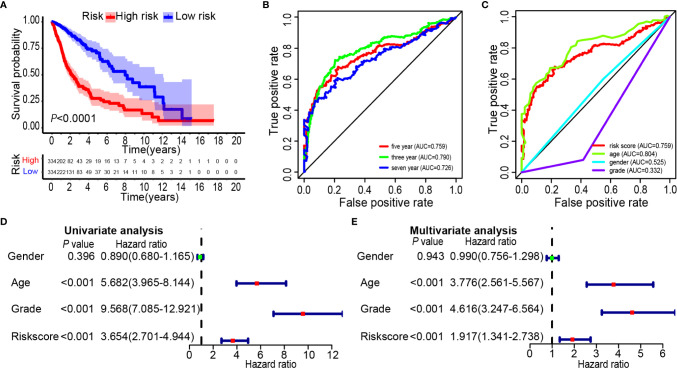
Prognostic significance of the 25-gene signature derived risk scores in TCGA cohort. **(A)** Kaplan-Meier analysis of TCGA gliomas patients was stratified by median risk. High-risk scores are associated with general poor survival of gliomas. **(B**, **C)** Multi-index ROC curve of risk score and other indicators. **(D)** Univariate Cox regression analysis. Forest plot of associations between risk factors and the survival of gliomas. **(E)** Multiple Cox regression analysis. The ferroptosis-related gene signature is an independent predictor of gliomas.

Glioma data from the TCGA was used to verify the risk score. The lasso regression analysis was performed on the TCGA data to calculate the patients’ risk scores using similar regression coefficients. Subsequently, KM survival analysis was used to assess the risk model. High-risk scores correlated with worse overall survival than the low-risk scores (**Figure 6A**). According to the results of ROC curve, we built a prediction model for predicting the overall survival patients (**Figures 6B, C**). Also, the prognostic value of the risk scores was evaluated. Univariate analysis revealed that the risk score was significantly correlated to the overall survival (HR = 3.654, 95% CI = 2.701–4.944, *P* < 0.001) in the TCGA LGG-GBM (**Figure 6D**). Multivariate analysis proved that the risk score was an independent prognostic indicator (HR = 1.917, 95% CI = 1.341–2.738, *P* < 0.001) (**Figure 6E**). The risk scores established with TCGA could classify glioma patients based on clinical features and could independently predict the prognosis of patients (**Table 3**). Patients in the high-risk group were characterized mainly by high-grade and older age (*P* < 0.001).

**Table 3:** Characteristics of patients in low and high-risk scores in TCGA cohort.

Characteristic	N	High-risk, N = 334 ^1^	Low-risk, N = 334 ^1^	*p*-value ^2^
**Age**	668			<0.001
≤41		105	175	
>41		229	159	
**Gender**	668			0.6
Female		138	145	
Male		196	189	
**Grade**	668			<0.001
WHO II		96	151	
WHO III		122	139	
WHO IV		116	44	

^1^Statistics presented: n.

^2^Statistical tests performed: chi-square test of independence.

### Individualized Prognostic Prediction Models

**Figure 7:**
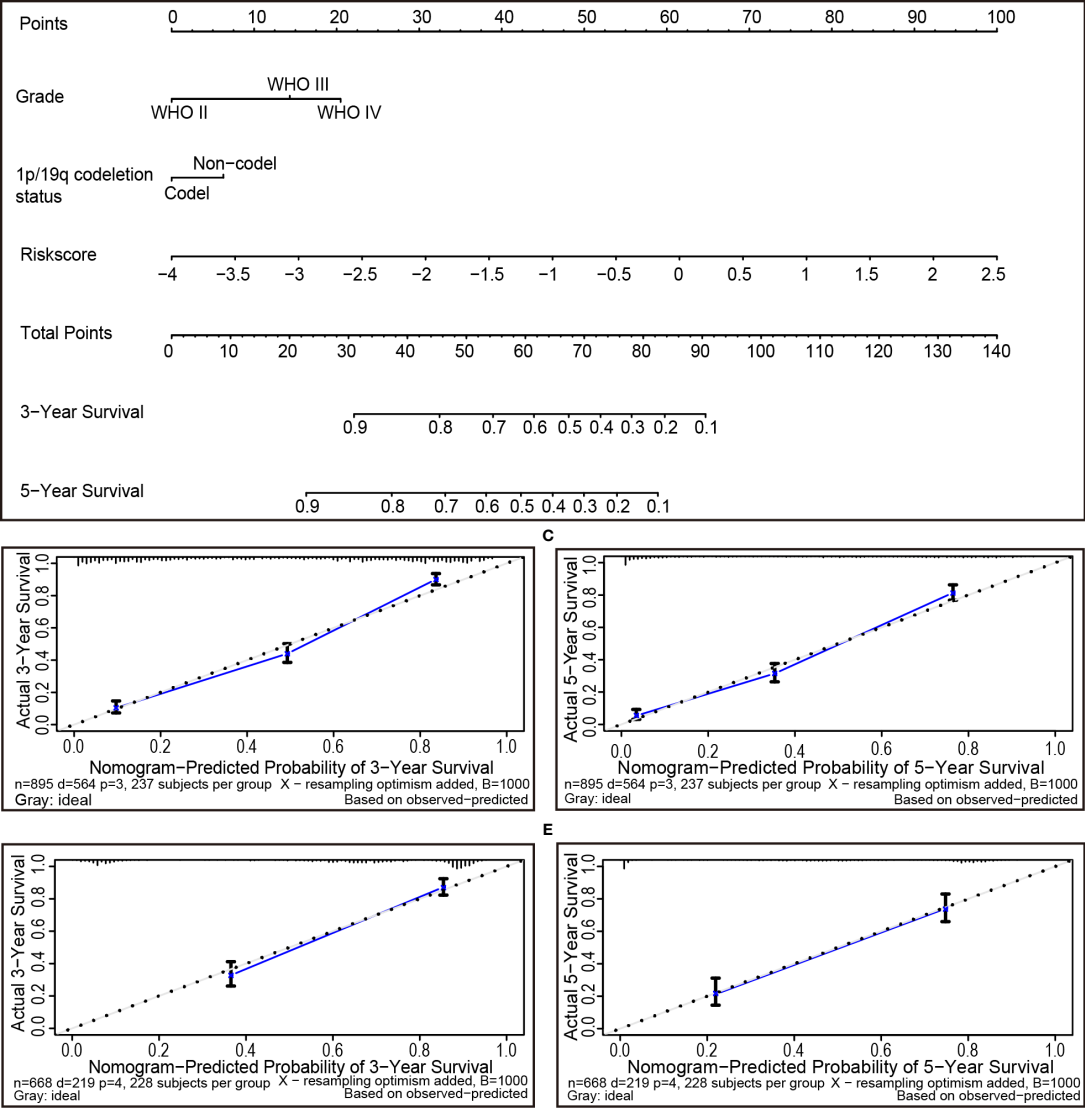
The nomogram can predict the prognosis probability in gliomas. **(A)** A nomogram of the gliomas cohort (training set) used to predict the overall survival. **(B, C)** Calibration maps used to predict the 3-year **(B)** and 5-year survival **(C)** in the training set. Calibration plots for 3-year **(D)** and 5-year survival **(E)** in the TCGA cohort (test group). The x-axis and y-axis represent the predicted and actual survival rates of the nomogram, respectively. The solid line represents the predicted nomogram, and the vertical line represents the 95% confidence interval.

During the quantification of the risk on individuals in a clinical setting with the integration of multiple risk factors, the nomogram acts as a powerful tool in the assessment. Using the synthesis of 25 ferroptosis-related gene signature, a nomogram was generated based on grade, 1p/19q codeletion status and risk score to predict the probability of 3 and 5-year overall survival rates. Meanwhile, the calculated C index was to be 0.789. Several factors were scored based on the proportion of contribution to survival risk as shown in **Figure 7A**. The calibration curve results showed that the predicted survival rate is closely related to the actual survival rate (**Figures 7B, C**). For instance, if a patient had stage IV (20 points), 1p/19q non-codeletion (6 points) and a risk score of 0.5 (69 points), cumulatively, her totals are 95, and her 3- and 5-year overall survival rates would both be zero. The nomogram was verified in the TCGA cohort. The 3- and 5-year calibration curves are displayed in **Figures 7D, E**, respectively.

### Analysis of Biological Properties and Pathways Related to the Gene Signatures

**Figure 8:**
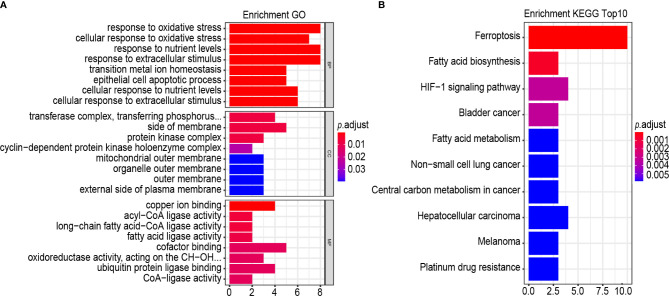
GO, and KEGG analysis. **(A, B)** Functional annotation of 25-gene using GO terms and KEGG pathway. GO **(A)** and KEGG **(B)**. “BP” stands for “biological process”, “CC” stands for “cellular component”, and “MF” stands for “molecular function”.

GO term and the KEGG pathway analyses were performed to annotate the biological characteristics of 25 gene signatures used to construct risk models. The main biological processes (BPs) involved include response to oxidative stress, cellular response to oxidative stress, response to nutrient levels, response to extracellular stimulus, transition metal ion homeostasis, epithelial cell apoptotic process, cellular response to nutrient levels and cellular response to extracellular stimulus (**Figure 8A**). The most abundant cellular component (CC) terminology was the transferase complex, transferring phosphorus-containing groups, side of membrane, protein kinase complex and cyclin–dependent protein kinase holoenzyme complex (**Figure 8A**). The most abundant molecule function (MF) term was copper ion binding, acyl-CoA ligase activity and long-chain fatty acid–CoA ligase activity, fatty acid ligase activity, cofactor binding, oxidoreductase activity, acting on the CH-OH group of donors, NAD or NADP as acceptor, ubiquitin protein ligase binding and CoA-ligase activity (**Figure 8A**). Results of the KEGG pathway analysis revealed that the most abundant pathways were ferroptosis and fatty acid biosynthesis (**Figure 8B**).

## Discussion

Ferroptosis, a novel form of cell death, completely differs from apoptosis, autophagy, and necrosis (17), and is characterized by unique morphology, gene expression, and molecular pathways. Previous studies identified that GSH, GPX4 activity inhibition, and iron-dependent ROS burst are the critical factors inducing ferroptosis (7). Small-molecule drugs have been shown to promote ferroptosis, for instance, erastin and RSL3 (18). Thus, ferroptosis inducers have the potential to treat tumors (19). Resistance of cancer cells to chemotherapy is a major problem in cancer therapy. Since the cell death process of ferroptosis is different from apoptosis, it can overcome the low efficiency of apoptosis-inducing chemical drugs in cell death induction. Hence, ferroptosis inducers may provide novel solutions to the tumor drug resistance problem (20). Also, ferroptosis pathway activation can induce the death of cancer cells, especially in the context of drug resistance, which can enhance the cancer sensitivity to chemotherapy drugs. Integrating ferroptosis inducer and chemotherapy in cancer treatment can attain a synergistic response, thereby promoting chemotherapy sensitivity. Compelling evidence reveals that the GPX4 inhibitors show some level of lethality in drug-resistant cells through ferroptosis, and targeting of GPX4 may be a therapeutic strategy for preventing acquired drug resistance (21). Besides, it has been shown that Cisplatin combined with erastin improves anti-tumor activity significantly, which reflects the importance of ferroptosis in tumor treatment (22). In addition, autophagy suppression in GBMs can enhance the sensitivity of GBM stem cells by inducing ferroptosis (11). Thus, combined therapy of TMZ and erastin may be an effective treatment for GBM, and targeted ferroptosis can be one of the potential therapies to reverse TMZ resistance (23).

In recent years, the promotion of anti-tumor immune response *via* immunotherapy has become a debatable one in the GBM treatment, and novel types of immunotherapy have proposed the possibility of transforming GBM, which is a “cold tumor”, into a “hot tumor” (24). Nevertheless, given the strong immunosuppression and immune evasion properties of GBMs, Anti-PD-1 (Nivolumab) immunotherapy is unable to improve the overall survival of patients with relapsed GBM, so that the GBM immunotherapy is faced with huge challenges (25). Interestingly, integrating immunotherapy with ferroptosis inducers had become a prospect. Research has shown that post after anti-PD-L1 treatment with immune checkpoint inhibitors, the level of ferroptosis-specific lipid peroxidation was elevated markedly, while blockage of ferroptosis pathway led to the significantly low sensitivity of tumor cells to immunotherapy. In addition, studies have found that the IFNγ secreted by activated CD8+T cells can inhibit the system X_C_¯ *via* the JAK1-STAT1 pathway, thereby regulate the development of ferroptosis (26). Moreover, a recent study revealed that fatty acid called dihomogamma-linolenic acid (DGLA) can kill fibrosarcoma HT1080 cells that are sensitive to ferroptosis. Results of the study demonstrated that nematodes are good animal models for studying ferroptosis (27). It is likely that ferroptosis presents a new therapeutic avenue for cancer patients, especially those with resistance to post-conventional chemoradiotherapy or to whom immunotherapy is ineffective.

In this study, we found for the first time that the ferroptosis-related genes can classify glioma patients into two classes, which exhibit significant differences in clinical and molecular features. Gene markers related to ferroptosis were established. Through LASSO regression analysis, the patients can be classified into high-risk and low-risk groups. In focussing the genetic diversity, we established signatures based on 25 genes, which comprised the protective genes (BAP1, GLS2, CISD1, PRNP, AKR1C3, TF, ACACA, ACSL6, and MAP3K5) and the risk-related genes (CDKN1A, G6PD, HSPB1, LOX, STEP3, ACSL1, CP, HMOX1, CYBB, ANO6, RB1, PCBP1, PGD, AURKA, G3BP1, and TP53). Therefore, patients with diffuse gliomas can be classified into low-risk and high-risk groups for discrimination of clinical outcomes.

These genes can be roughly ranged into four categories, including iron metabolism (CISD1, PRNP, HSPB1, PCBP1, STEAP3, TF, and CP), lipid metabolism and (anti)oxidant metabolism (ACACA, ACSL1, ACSL6, AKR1C3, LOX, MAP3K5, HMOX1, ANO6, and RB1), energy metabolism (G6PD, PGD, CYBB, and GLS2), and cancer metabolism (BAP1, AURKA, CDKN1A, G3BP1, and TP53) (12). In terms of iron metabolism, CISD1 limits mitochondrial iron uptake and inhibits ferroptosis by protecting mitochondrial lipid peroxidation. Altered in metal homeostasis is thought to be associated with many neurodegenerative diseases (28). The expression level of prion protein (PRNP) changes the content of copper, iron and zinc in specific brain regions of mice (29). HSPB1 phosphorylation negatively regulates the occurrence of ferroptosis by reducing cellular iron uptake and the production of lipid reactive oxygen species (30). As the chaperone of intracellular iron, PCBP1 can control the redox activity of labile iron pool, thus inhibiting ferroptosis (31). As a metalloreductase, STEAP3 regulates cellular iron uptake and homeostasis and promotes the malignant progression of gliomas (32). Transferrin (TF) and ceruloplasmin (CP) also play a vital role in ferroptosis (33, 34). In terms of lipid metabolism and (anti)oxidant metabolism, ACC1, a lipid synthase encoded by ACACA, is a rate-limiting enzyme in fatty acid synthesis. The decrease in its activity can inhibit drug-induced ferroptosis (35). ACSL1 and ACSL6 are members of the long-chain acyl-CoA synthase family. ACSLs generally make cancer cells resistant fatty acid-induced lipotoxicity and cell death (36). Interestingly, ACSL4 can induce ferroptosis through oxidized arachidonic acid (37). In gliomas, ACSL4 can inhibit the proliferation of tumor cells by activating ferroptosis (38). AKR1C family members can regulate the detoxification of lipid oxide decomposition products, and the expression of AKR1C family members increases sharply in drug-resistant cell lines of erastin. Its overexpression can enhance the detoxification of reactive aldehydes produced downstream of oxidative destruction of the plasma membrane during the formation of ferroptosis, resulting in partial resistance to erastin (39). The expression of AKR1C was increased in TMZ-resistant glioma cell lines (40). Therefore, whether AKR1C can simultaneously mediate TMZ resistance and inhibit ferroptosis in gliomas is worthy of further study. Cu-dependent lysyl oxidase (LOX) is closely related to ROS (41). MAP3K5 (ASK1) is downstream of lipid peroxides, and erastin can activate the ASK1-p38 axis, resulting in ASK1-dependent ferroptosis (42). As a dual regulator of iron and ROS homeostasis, HMOX1 plays a leading role in ferroptosis (43). The activation of ANO6 (TMEM16F) can destroy the stability of the plasma membrane, resulting in cell ferroptosis (44). The negative state of RB1 protein can promote the occurrence of ferroptosis in hepatoma cells induced by sorafenib (45). In terms of energy metabolism, G6PD and PGD, associated with the pentose phosphate pathway, can prevent erastin-induced ferroptosis when it is knocked down in non-small cell lung cancer cells. NADPH oxidase CYBB (NOX2) is one of the sources of ROS, and NOX inhibitors can strongly inhibit ferroptosis induced by erastin (3). Glutaminase 2 (GLS2) can convert glutamine to glutamate, and knockdown GLS2 can inhibit ferroptosis induced by erastin or amino acid/cystine deprivation (33). In terms of cancer metabolism, the tumor suppressor BRCA1-associated protein-1 (BAP1) inhibits SLC7A11 expression in a de-ubiquitin-dependent manner, resulting in lipid peroxidation and ferroptosis (46). In upper gastrointestinal adenocarcinoma, inhibition of AURKA can reduce the expression of GPX4 and induce cell death. Furthermore (47), AURKA inhibitors may be effective in the treatment of hepatocellular carcinoma with TP53 mutation (48). Ras-GTPase-activating protein-binding protein 1 (G3BP1) is relevant in a variety of carcinogenic signaling pathways such as TP53 and RAS. The interaction between lncRNA P53RRA and G3BP1 makes TP53 trapped in the nucleus, resulting in cell cycle arrest, apoptosis, and ferroptosis (49). TP53 stabilization can inhibit the ferroptosis of cancer cells by inducing the expression of the TP53 transcriptional target gene CDKN1A (P21) (50). Besides, TP53 enhanced ferroptosis by targeting SLC7A11, GLS2, or SAT1 (51, 52). On the contrary, TP53 can also inhibit ferroptosis by targeting DPP4 (53). This suggests that TP53 plays a dual and context-dependent role in the regulation of lipid peroxidation in cancer ferroptosis (54). TP53 signaling has complex mechanisms in ROS-mediated ferroptosis through transcriptional and non-transcriptional metabolic targets (55). It is essential to further study the relationship between TP53 and ferroptosis in glioma. In short, a large number of previous studies have shown that these 25 genes are closely associated with ferroptosis, which provides an important theoretical basis for our risk model based on ferroptosis-related genes.

Moreover, with the inclusivity of some clinical and molecular features, we demonstrated that the risk score of ferroptosis-related genes is an independent prognostic indicator of overall survival for patients with diffuse gliomas. In 2016 WHO classification, codeletion of chromosomal arms 1p/19q (1p/19q codeletion) and isocitrate dehydrogenase 1 or 2 (IDH1 or IDH2) were included in diagnostic typing for glioma classification (56). Excitingly, the risk score was also an independent prognostic indicator in patients with different molecular types of gliomas. Herein, the 25 gene risk signatures exhibited greater prognostic value than the conventional factors such as “age”, “grade”, and “IDH status”. This indicates that the gene expression-based signatures can predict the prognosis of gliomas better. The risk model built with CGGA was verified using risk scores by survival analysis and univariate analysis, multivariate Cox analyses on the TCGA data, where the patients’ risk scores were calculated using a similar regression coefficient as CGGA. The risk scores established with TCGA showed significant clinical differences between two risk groups, and can independently predict the prognosis of gliomas. From the analysis outputs, it is suggestive that the risk score feature is a powerful prognostic indicator, which can be adopted for classifying patients and guiding targeted therapy in the future. Thereafter, we built individualized prognostic prediction models by utilizing nomograms were developed where the risks of individuals in the clinical context were quantified by integrating multiple risk factors. Calibration curves revealed high consistency between the actual and predicted overall survival rates.

Concerning, annotations, they have been developed to the BPs and pathways associated with this risk scores. The GO BP involved mainly includes the response to oxidative stress and cellular response to oxidative stress. The enriched KEGG pathways are ferroptosis and fatty acid biosynthesis. As observed, our study strengths include the systematic expression profile analysis, robustness of risk scoring method, as well as the validation across multiple platforms among multiple populations. Despite the confirmation of the predictive value of the 25 gene signatures in various datasets, larger-sample prospective studies are still needed to assess their clinical relevance. Besides, it is undeniable that compared with ferroptosis, some genes in the signature may be more strongly related to other pathways in gliomas. In summary, our results demonstrate that the 25 gene markers may be potential prognostic biomarkers providing new insight into the research and gliomas treatment.

## Conclusions

This study demonstrates that the ferroptosis-related genes can be used to classify glioma patients based on different clinical and molecular features. A risk score based on the 25 genes associated with the pathological features of gliomas is presented which can independently predict the prognosis of glioma patients. Moreover, this study provides a new understanding of ferroptosis in gliomas’ development and progression, and offers important ideas for developing ferroptosis promoters for the treatment of glioma. Given that our results are based on RNA-seq technology, further research is needed to explore the prognostic value of the proposed 25 gene marker.

## Data Availability Statement

The original contributions presented in the study are included in the article/**Supplementary Material**. Further inquiries can be directed to the corresponding authors.

## Author Contributions

KY and JT conceived and designed the study. SZ and ZC provided equal contributions to research design, data analysis and article writing. YY and JZ revised the manuscript. All authors contributed to the article and approved the submitted version.

## Funding

This work was funded by Hainan Provincial Key Research and Development Program Project Fund (ZDYF2019129) and the National Nature Science Foundation of China(82060456).

## Conflict of Interest

The authors declare that the research was conducted in the absence of any commercial or financial relationships that could be construed as a potential conflict of interest.
