# Supplementary material for: Clinical and Biological Significances of a Ferroptosis-Related Gene Signature in Glioma
Source: Front Oncol. 2020 Nov 20;10:590861. doi: 10.3389/fonc.2020.590861 (PMC7718027; doi:10.3389/fonc.2020.590861)
Supplement: Supplementary file 1 [file DataSheet_1.docx]

Supplementary Material

# Supplementary Figures


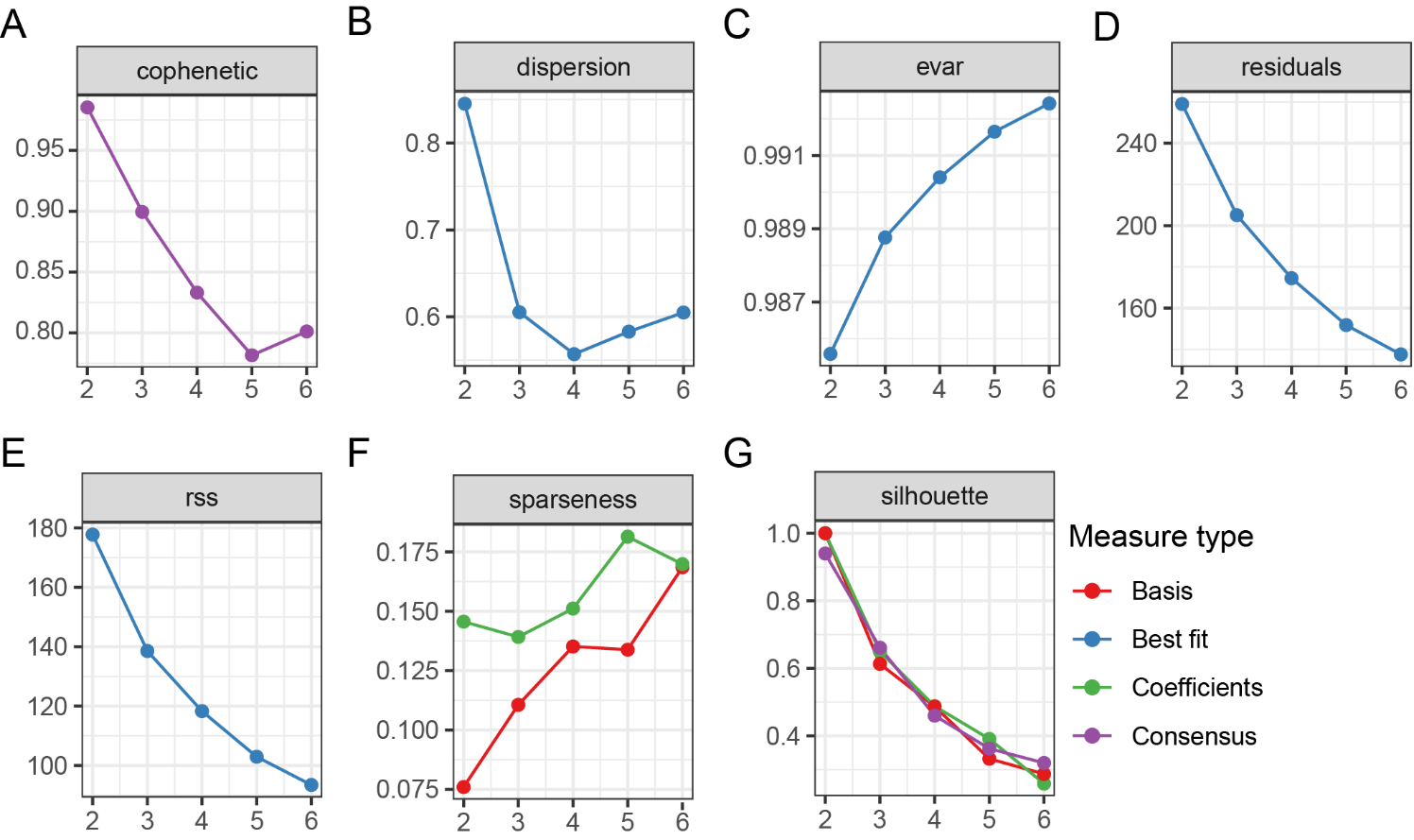


**Figure S1.** The relationship between cophenetic (A), dispersion (B), evar (C), residuals (D), rss (E), sparseness (F), and silhouette (G) coefficients concerning the number of clusters.


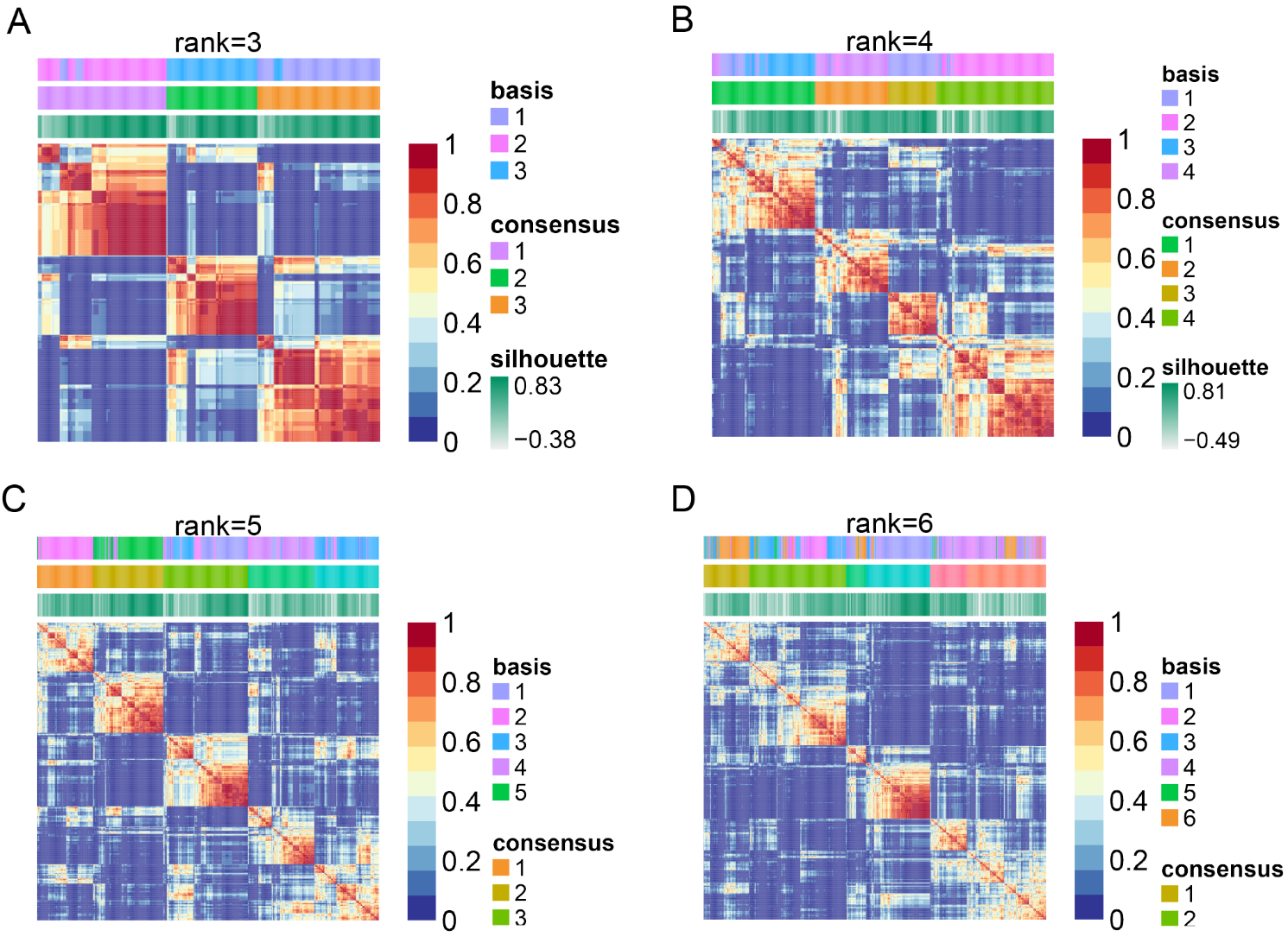
 **Figure S2.** Consensus matrix of NMF clustering for k = 3 (A),4 (B),5 (C) and 6 (D) in CGGA cohort.


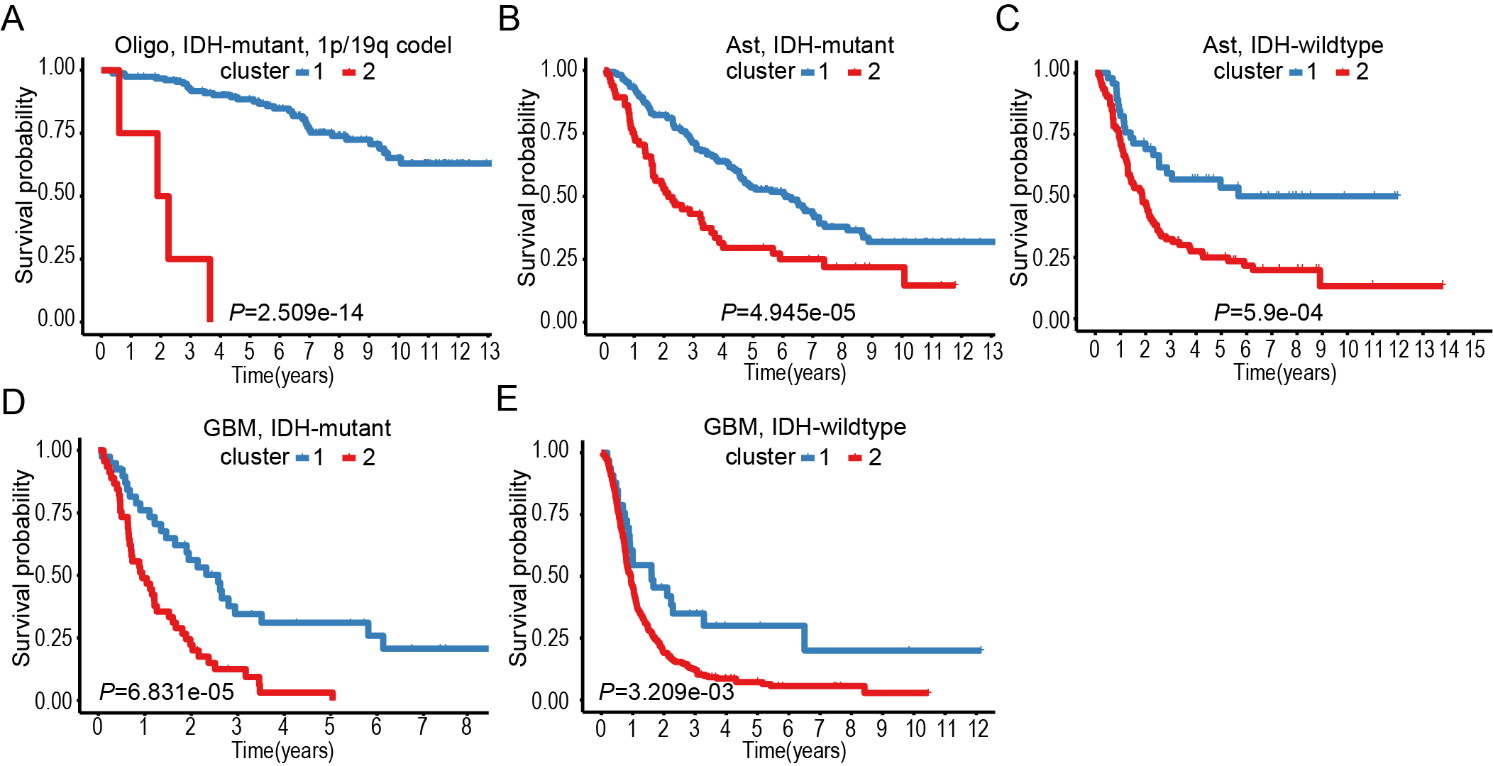


**Figure S3.** Prognostic value of the clusters in patients stratified by the integrated analysis of WHO 2016. (A-E) Kaplan–Meier overall survival curves for patients with Oligodendroglioma with IDH-mutant and 1p/19q co-deletion (A), Astrocytoma with IDH-mutant (B), Astrocytoma with IDH-wildtype (C), GBM with IDH-mutant (D), and GBM with IDH- wildtype (E).


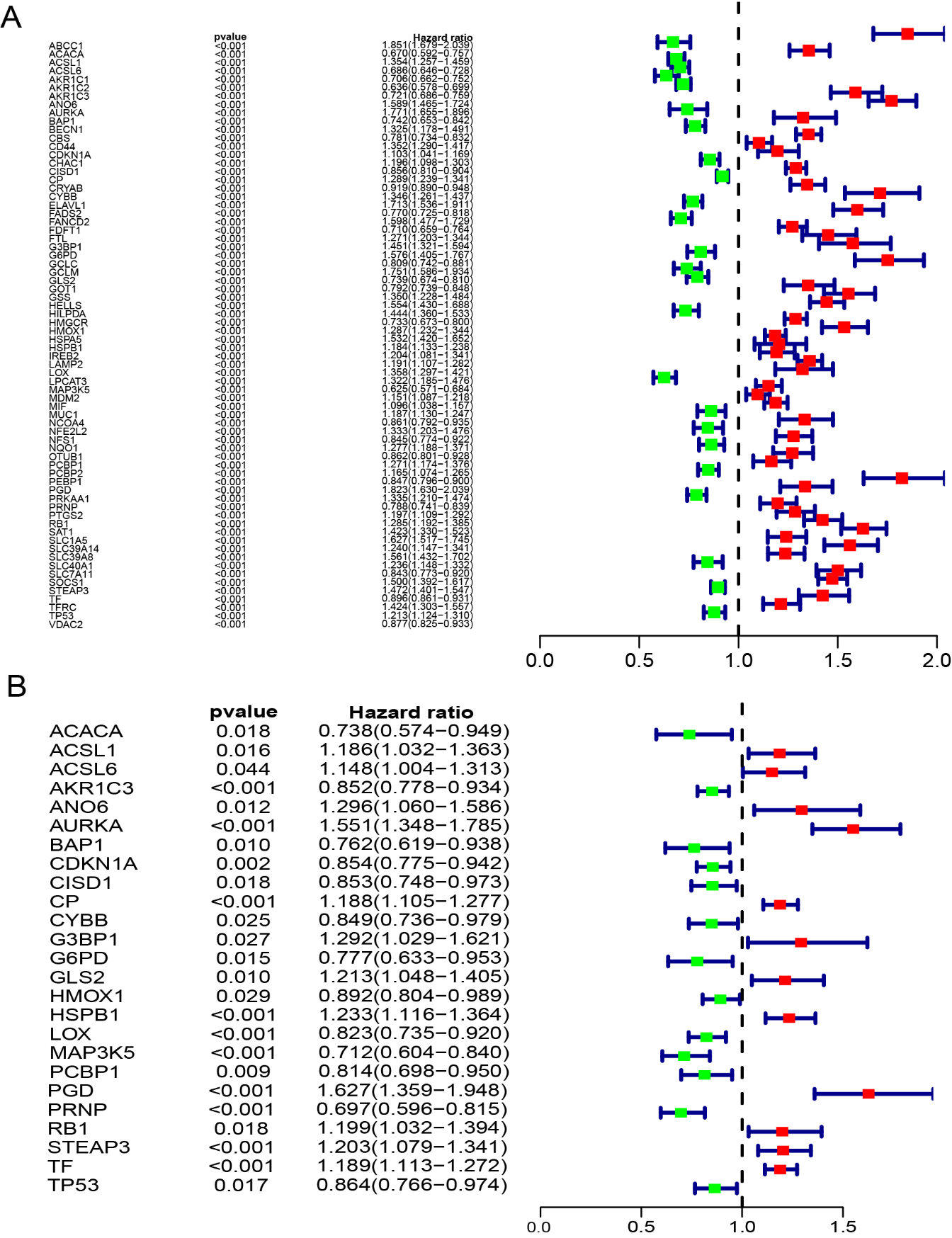


**Figure S4.**  Selection of ferroptosis genes associated with the survival of glioma by univariate Cox regression analysis and multiple Cox regression analysis. (A) univariate Cox regression analysis (B) multiple Cox regression analysis.


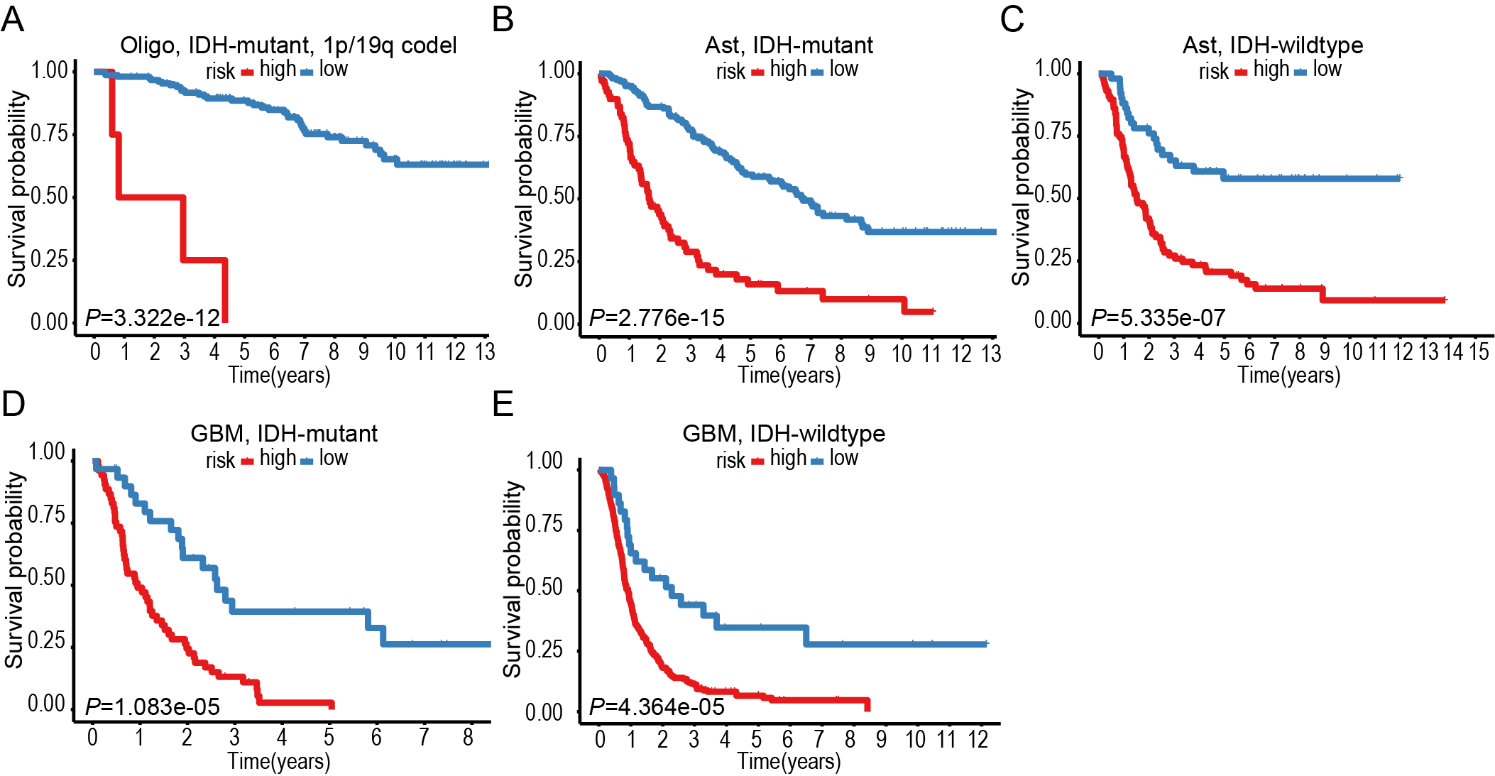


**Figure S5.** Prognostic value of the risk signature in patients stratified by the integrated analysis of WHO 2016. (A-E) Kaplan–Meier overall survival curves for patients with Oligodendroglioma with IDH-mutant and 1p/19q co-deletion (A), Astrocytoma with IDH-mutant (B), Astrocytoma with IDH-wildtype (C), GBM with IDH-mutant (D), and GBM with IDH- wildtype (E).


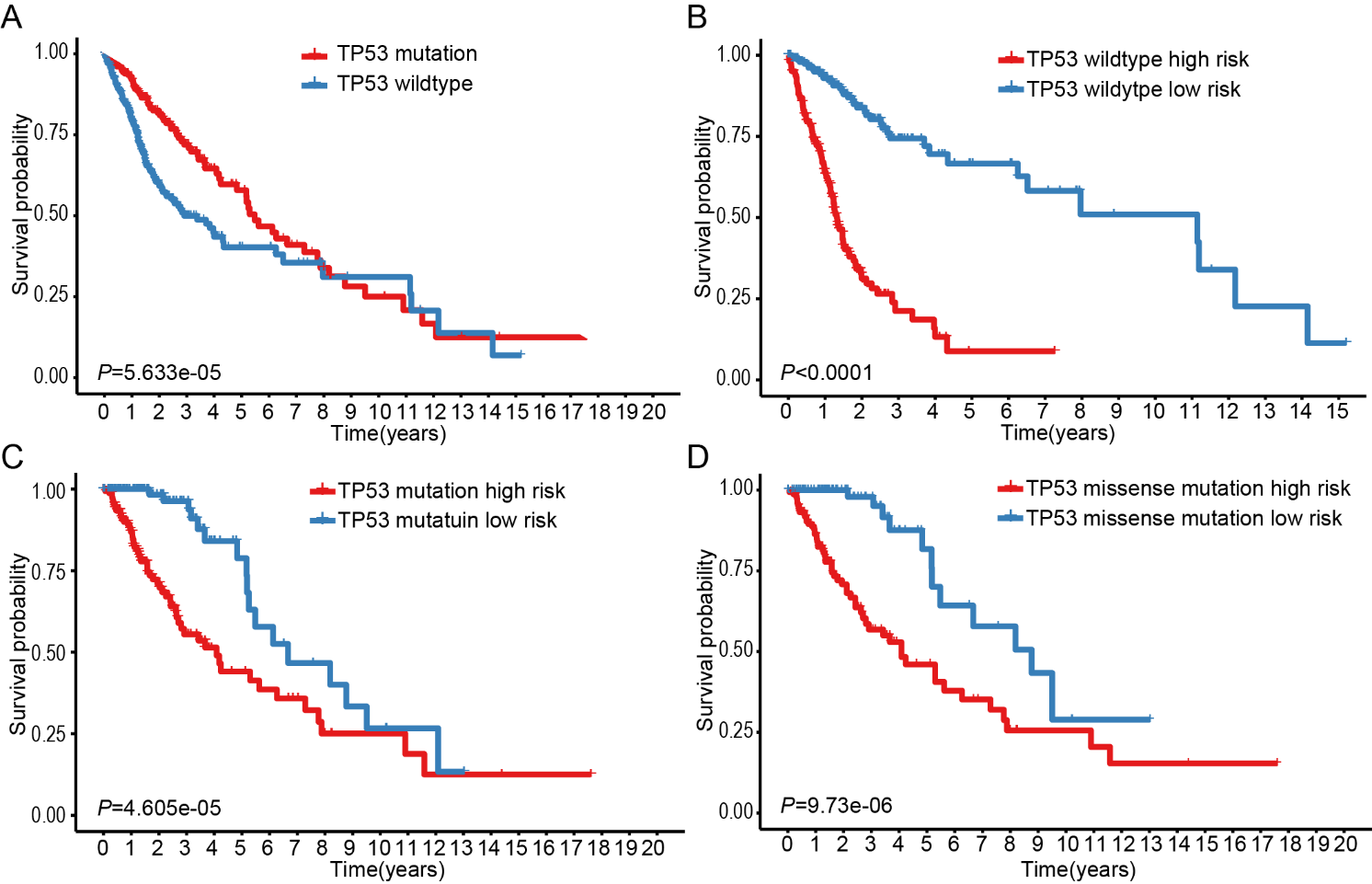


**Figure S6.** Kaplan-Meier analysis of overall survival according to TP53 mutation status Kaplan-Meier survival by TP53 status (A); in the TP53 mutation subgroup (B); in the TP53 wild-type subgroup (C); and in the TP53 missense mutation subgroup (D).


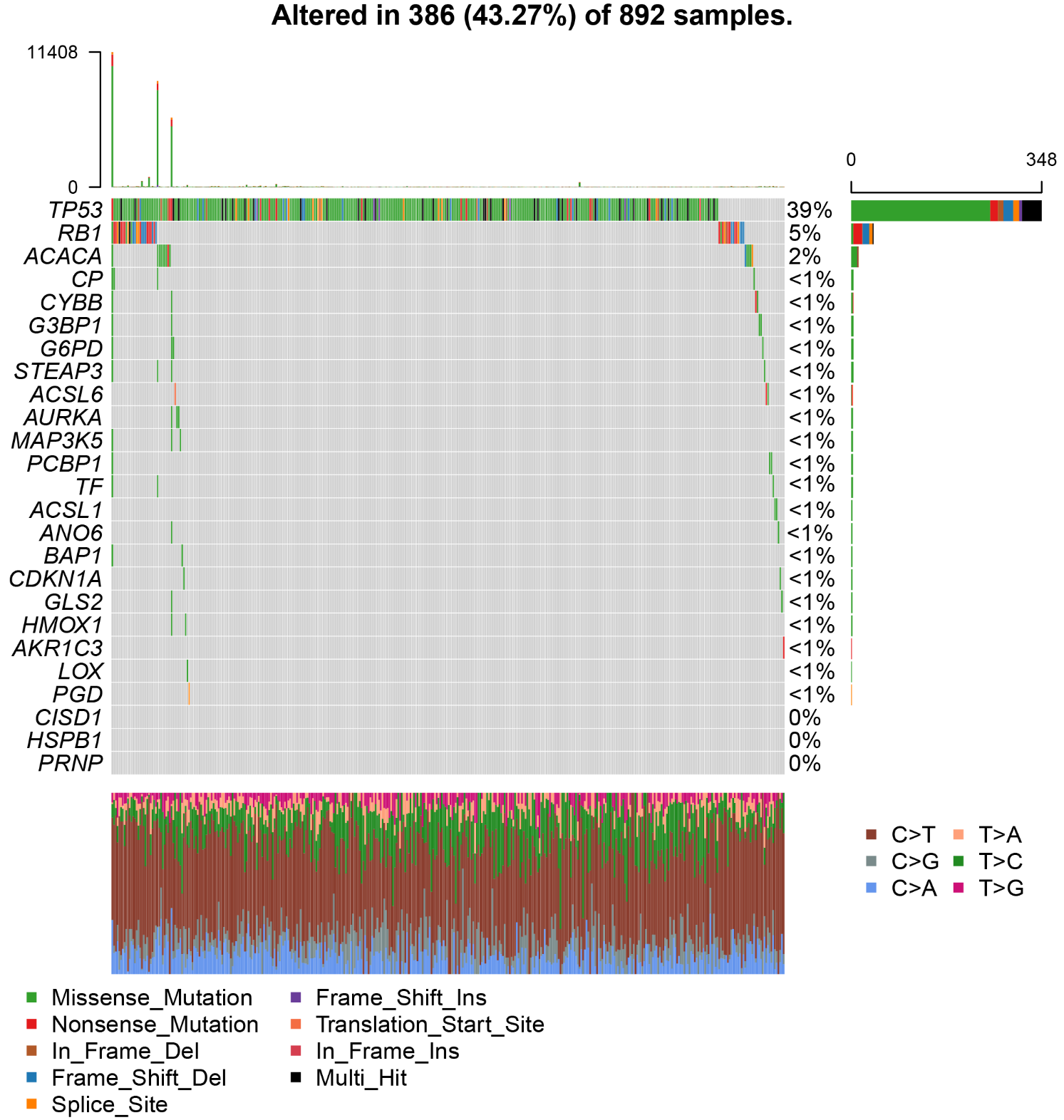


**Figure S7**. Genetic changes of the 25-gene in the gliomas from the TCGA dataset.


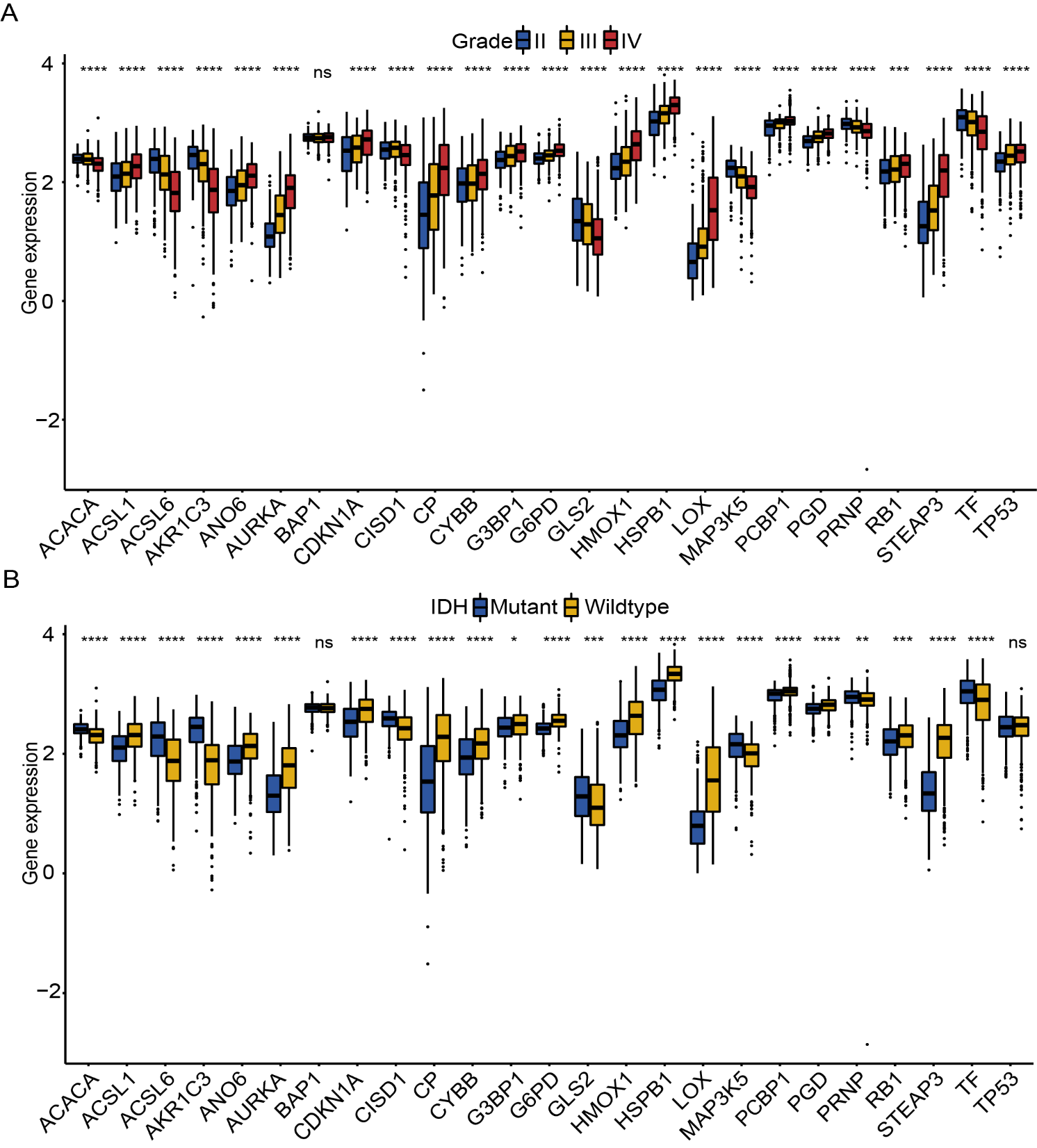


**Figure S8.** Expression of 25-gene in gliomas with different clinicopathological features.

# Supplementary Table

**Table S1**.the 113 ferroptosis related genes were included in this study.

| **Gene** | **Entrez ID** | **Gene** | **Entrez ID** | **Gene** | **Entrez ID** |
| --- | --- | --- | --- | --- | --- |
| ABCC1 | 4363 | EPAS1 | 2034 | MUC1 | 4582 |
| ACACA | 31 | FADS2 | 9415 | MYC | 4609 |
| ACO1 | 48 | FANCD2 | 2177 | NCOA4 | 8031 |
| ACSF2 | 80221 | FDFT1 | 2222 | NFE2L2 | 4780 |
| ACSL1 | 2180 | FH | 2271 | NFS1 | 9054 |
| ACSL3 | 2181 | FTH1 | 2495 | NQO1 | 1728 |
| ACSL4 | 2182 | FTL | 2512 | OTUB1 | 55611 |
| ACSL5 | 51703 | G3BP1 | 10146 | PCBP1 | 5093 |
| ACSL6 | 23305 | G6PD | 2539 | PCBP2 | 5094 |
| AKR1C1 | 1645 | GCLC | 2729 | PEBP1 | 5037 |
| AKR1C2 | 1646 | GCLM | 2730 | PGD | 5226 |
| AKR1C3 | 8644 | GLS2 | 27165 | PHKG2 | 5261 |
| ALOX12 | 239 | GOT1 | 2805 | PRKAA1 | 5562 |
| ALOX15 | 246 | GPX4 | 2879 | PRKAA2 | 5563 |
| ALOX15B | 247 | GSS | 2937 | PRNP | 5621 |
| ANO6 | 196527 | HELLS | 3070 | PTGS2 | 5743 |
| ATF4 | 468 | HILPDA | 29923 | RB1 | 5925 |
| ATG5 | 9474 | HMGB1 | 3146 | RPL8 | 6132 |
| ATG7 | 10533 | HMGCR | 3156 | SAT1 | 6303 |
| ATP5G3 | 228033 | HMOX1 | 3162 | SAT2 | 112483 |
| AURKA | 6790 | HSBP1 | 3281 | SLC11A2 | 4891 |
| BAP1 | 8314 | HSPA5 | 3309 | SLC1A5 | 6510 |
| BECN1 | 8678 | HSPB1 | 3315 | SLC39A14 | 23516 |
| CBS | 875 | IREB2 | 3658 | SLC39A8 | 64116 |
| CD44 | 960 | ITGA6 | 3655 | SLC3A2 | 6520 |
| CDKN1A | 1026 | KEAP1 | 9817 | SLC40A1 | 30061 |
| CDKN2A | 1029 | LAMP2 | 3920 | SLC7A11 | 23657 |
| CFTR | 1080 | LINC00472 | 79940 | SOCS1 | 8651 |
| CHAC1 | 79094 | LOX | 4015 | SQLE | 6713 |
| CISD1 | 55847 | LPCAT3 | 10162 | STEAP3 | 55240 |
| CP | 1356 | MAP1LC3A | 84557 | TF | 7018 |
| CRYAB | 1410 | MAP1LC3B | 81631 | TFRC | 7037 |
| CS | 1431 | MAP1LC3C | 440738 | TP53 | 7157 |
| CYBB | 1536 | MAP3K5 | 4217 | TP63 | 8626 |
| DPP4 | 1803 | MAPK1 | 5594 | VDAC2 | 7417 |
| EGLN1 | 54583 | MDM2 | 4193 | VDAC3 | 7419 |
| ELAVL1 | 1994 | MIF | 4282 | ZEB1 | 6935 |
| EMC2 | 9694 | MT1G | 4495 |  |  |
